# Sustained Platelet Improvement Temporally Associated with Dupilumab in Chronic Immune Thrombocytopenia: A Case Study

**DOI:** 10.3390/jcm15197539

**Published:** 2026-09-28

**Authors:** Xinyu Xu, Nicholas R. Burwick, Sarah K. Baxter, Aaron B. Boothby, Vincent S. Fan, Jesse J. Salk

**Affiliations:** 1 Department of Pharmacy, VA Puget Sound Healthcare System, Seattle, WA 98108, USA; 2 Department of Medicine, Divisions of Hematology, Oncology and Bone Marrow Transplantation, VA Puget Sound Healthcare System, Seattle, WA 98108, USA; 3 Department of Medicine, Division of Hematology and Oncology, School of Medicine, University of Washington, Seattle, WA 98195, USA; 4 Zag Bio, Cambridge, MA 02139, USA; 5 Department of Medicine, Division of Pulmonary and Critical Care, VA Puget Sound Health Care System, Seattle, WA 98108, USA; 6 Department of Medicine, Division of Pulmonary, Critical Care, & Sleep Medicine, School of Medicine, University of Washington, Seattle, WA 98195, USA; 7 Clinical Research Division and Translational Science and Therapeutics Division, Fred Hutch Cancer Center, Seattle, WA 98109, USA

**Keywords:** ITP, IL-4 receptor antagonist, TPO-RA-sparing, Veterans Affairs, case study

## Abstract

**Introduction:** Immune thrombocytopenia (ITP) is an autoimmune disorder characterized by reduced platelet counts. Dupilumab, an IL-4 receptor antagonist, is effective in allergic and auto-inflammatory diseases; however, use in ITP remains unexplored. **Case Presentation:** A 78-year-old male with chronic ITP, maintained on a thrombopoietin receptor agonist (TPO-RA) with an average platelet count of around 78,000/μL, demonstrated a sustained rise in platelet counts (>100,000/μL) shortly following initiation of dupilumab for an unrelated chronic obstructive pulmonary disease. This improvement enabled a stepwise reduction in TPO-RA to <20% of his base dosage while maintaining adequate platelet levels for three years since. Despite detailed patient interviews and chart review, we were unable to identify any clinical factors besides commencement of dupilumab as a potential explanation. If indeed causative, we speculate that a TPO-RA-sparing effect of dupilumab might occur via disruption of IL-4 signaling, leading to inhibited Th9 cell activation and subsequent IL-21 production to restore Treg/Th17 balance, thus contributing to decreased platelet destruction. **Discussion:** This case supports a potential role for dupilumab in the treatment of chronic ITP. To our knowledge, it is the first report suggesting a therapeutic benefit of IL-4 blockade in this disease. Identification of predictive immunologic biomarkers, such as cytokine profiles or T-cell subset signatures, might help identify patients most likely to benefit from IL-4 pathway modulation. Further controlled studies are warranted to clarify the immunologic mechanisms underlying this response and to assess the therapeutic potential of targeting the IL-4 axis in ITP.

## 1. Introduction

Immune thrombocytopenia (ITP) is a rare autoimmune bleeding disorder characterized by low platelet levels (<100,000/μL), resulting from antiplatelet antibodies, T-cell-mediated platelet destruction, and impaired platelet production [1]. It affects both adults and children; in adults, the prevalence of primary ITP is about 9.5 per 100,000, and the incidence is around 3.3 per 100,000 per year [2].

The canonical pathophysiologic mechanism of ITP involves macrophages phagocytosing platelets and subsequently presenting platelet-derived antigens, which then activate CD4+ T cells. These T cells stimulate B cells that later differentiate into antibody-producing plasma cells. Phagocytes then recognize and opsonize antibody-bound platelets, leading to a consumptive thrombocytopenia. An imbalance of Treg/Th17 ratio is often observed in ITP, which is associated with dysregulated autoimmune cell populations [2,3].

First-line treatment for severe ITP includes glucocorticoids and intravenous immunoglobulin (IVIG). Thrombopoietin receptor agonists (TPO-RA), such as eltrombopag, avatrombopag, and romiplostim, are now commonly used in the chronic setting to stimulate platelet production. Other treatments include rituximab, fostamatinib, rilzabrutinib, and splenectomy for patients who fail other therapies [2,3,4]. There remains a need for both additional therapies and better biomarkers to guide selection thereof in patients with relapsed/refractory ITP.

Dupilumab is a monoclonal antibody that inhibits the interleukin-4 (IL-4) pathway by binding to the type I interleukin-4 receptor (IL-4R) [5]. It is approved for treatment of inflammatory conditions including asthma, chronic obstructive production disease, and atopic dermatitis. While its use in the management of ITP remains largely unexplored, at least a potential relevance is suggested by the fact that certain genetic polymorphisms in IL-4 have been associated with the severity of ITP and the degree of TPO-RA requirements [6,7]. As far as we can tell, the case described herein is the first published report of an observed temporal association between the initiation of an IL-4R antagonist and a strong sustained platelet response in the setting of ITP being managed with a TPO-RA. This case report was prepared in accordance with CARE guidelines (Supplementary Table S1).

## 2. Case

This is a case of a 78-year-old male patient living with ITP for more than ten years who had a platelet level of 13,000/μL at the time of initial diagnosis. His pre-diagnosis baseline platelet count ranged from 50,000 to 145,000 within our record system, and he had no clinical signs of bleeding or bruising. The patient’s original workup for ITP included pertinent negatives of any prior history of transfusions, intravenous drug, or significant alcohol consumption within the decade prior to diagnosis. Serologic testing confirmed the absence of hepatitis C (HCV), human immunodeficiency virus (HIV), or H. pylori infection. His other medical history included chronic obstructive pulmonary disease (COPD) from past (but not current) smoking, major depressive disorder (MDD), generalized anxiety disorder (GAD), well-controlled hypothyroidism, and obstructive sleep apnea (OSA). Bone marrow biopsy in 2014 showed no signs of dysplasia or other abnormalities to suggest myelodysplastic syndrome (MDS); chronic lymphocytic leukemia (CLL/SLL); multiple myeloma; monoclonal gammopathy of undetermined significance (MGUS); or other lymphoid, myeloid, or marrow-infiltrative disorders. Renal and hepatic function were within normal limits, as were peripheral red cell and white cell counts. Although antiplatelet antibody testing was not performed, after excluding all the other secondary causes of ITP, the clinical diagnosis of chronic immune-mediated ITP was supported by brisk steroid responsiveness. Following initial treatment with steroids, the patient transitioned to eltrombopag daily in 2014.

**Figure 1:**
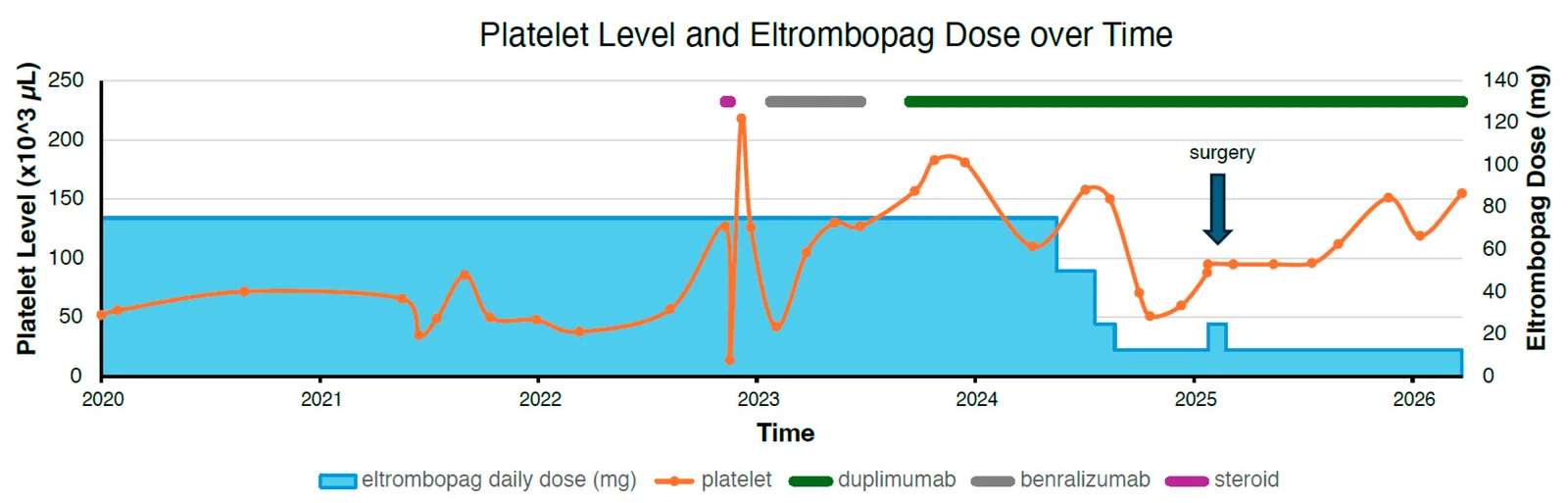
Platelet count over time in relation to eltrombopag dose and the initiation of dupilumab. The patient was maintained on eltrombopag 75 mg daily from early 2020, with fluctuating platelet levels averaging ~78,000/μL. In late 2023, dupilumab was initiated and platelet levels rose steadily for several months. Subsequently, eltrombopag was tapered: first to 50 mg, then 25 mg, and finally to 12.5 mg. Eltrombopag was temporarily increased to 25 mg as a perioperative precaution (early 2025) and then reverted to 12.5 mg daily. There was no steroid exposure as of a COPD exacerbation in 2023, including after dupilumab initiation.

The patient’s ITP was stable on a dose of eltrombopag 75 mg PO daily from early 2020 through the autumn of 2023. His platelet level fluctuated but averaged around 78,000 per μL (Figure 1 and Table 1). A notable high outlier value of 218,000 was observed in early 2023, which corresponded to a short course of steroids prescribed for COPD exacerbation and bronchitis, whereafter platelets returned to baseline. Starting in late 2023, the patient was noted to have sustained, uncharacteristically high platelet levels ranging from 157,000 to 183,000 over a three-month period. In the middle of 2024, given platelet counts consistently above 100,000, eltrombopag was reduced to 50 mg daily. Two months later, the patient continued to have platelet levels exceeding 150,000. Eltrombopag was further reduced to 25 mg daily in late summer of 2024 and still achieved a platelet level of 150,000 after this dose change. Shortly after, eltrombopag was lowered to 12.5 mg daily in early fall. Despite being on the lowest feasible (in terms of tablet size) dose of eltrombopag, the patient’s platelet count remained above 50,000, averaging 73,000 for five months from early fall to spring of 2025. At the beginning of 2025, holding eltrombopag was considered, but the patient underwent a pulmonary wedge resection for a stage I non-small cell lung cancer that was identified on surveillance CT imaging in early 2025. As a precaution, his eltrombopag was not held and was briefly increased to 25 mg daily to ensure a platelet count above 70,000, at the request of the patient’s thoracic surgery team, in late spring of 2025 for one month. Post-surgery eltrombopag was again reduced to the 12.5 mg dose, with platelets remaining above 95,000 for one year as of late spring of 2025. The option to discontinue eltrombopag was considered again after surgical recovery, but the patient’s preference was to continue taking the medication in anticipation of a possible future orthopedic procedure.

During the time of sequential eltrombopag dose reduction from late fall of 2023 through late spring of 2026, based on the patient’s reporting and fill history, he was adherent to the eltrombopag regimen. No meaningful changes to the patient’s medical problem list were noted from his chart. The timing of lab draws and the processing lab during this period was the same as throughout his prior ITP treatment. Other possible secondary causes of ITP were reassessed, including HIV and HCV, both of which the patient tested negative for on multiple occurrences. His white blood cell count was always in normal range, except for a brief spike after steroid exposure during a COPD exacerbation, and his hematocrit stably remained between 33% and 37% with stably normal mean corpuscular volume (MCV). Liver and renal function labs were also normal and unchanged from baseline. The patient had modest weight loss in late 2024 while on 12.5 mg eltrombopag; this returned to baseline in 2025 without any dose changes. He denied any meaningful change in diet during this period, and his serum albumin remained stable within normal limits.

One significant change noted, however, was that the patient began taking two new medications near the beginning of this period. He received monthly 30 mg doses of benralizumab (an IL-5 receptor antagonist) for asthma from early 2023 through mid-2023 (total of 5 doses.) Platelet levels remained steady during this course. After stopping this for lack of efficacy, he started on the IL-4 receptor antagonist (IL-4 RA), dupilumab. He received an initial 400 mg loading dose of dupilumab and began 200 mg biweekly maintenance doses thereafter. It proved quite effective for managing his COPD and asthma, and he has remained on it ever since. At the first lab check following the initial dupilumab dose, his platelets rose above 150,000 for the first time in multiple years (besides an isolated spike in 2023 following a steroid exposure). As shown in Figure 1, this remained elevated for several subsequent lab checks, which facilitated eltrombopag taper. The patient did not take any steroids, rituximab, or other ITP-directed treatments during the 10 months before or any time after starting dupilumab.

## 3. Discussion

To our knowledge, this is the first report of a sustained platelet response temporally associated with dupilumab in a patient with ITP based on a literature search in PubMed, Embase, and Medline from inception to June 2025 using terms “[dupilumab]” or “[benralizuamb]” and “[ITP]”. In this patient, dupilumab provided not only improvement to the patient’s pulmonary condition but also precisely coincided the unexpected platelet count improvement that allowed a clinically meaningful reduction (>80%) of his baseline dose of eltrombopag.

Dupilumab is a monoclonal antibody that binds to the alpha subunit of the IL-4 receptor (IL-4Rα), blocking the signaling of IL-4 and IL-13 cytokine-induced inflammatory responses [6]. The exact role of IL-4 and its receptor in ITP is not well characterized, and no IL-4R antagonists are currently used to treat ITP. Prior to the initiation of dupilumab, our patient had been trialed on benralizumab, which may have been accompanied by a transient rise—but not a sustained increase—in platelet count. Benralizumab has a different mechanism of action from dupilumab and binds to the alpha subunit of the IL-5 receptor, suggesting that a putative effect on platelets by dupilumab is specific to inhibition of the IL-4 pathway [8].

IL-4 is a cytokine that interacts with the IL-4 receptor (IL-4R) leading to downstream immunomodulatory effects, including polarization of T cells towards a Th2 phenotype and supporting B-cell growth and differentiation. The type I IL-4 receptor consists of IL-4Rα paired with the common gamma chain and is primarily found on hematopoietic cells. IL-4 supports immunoglobulin class switching, promoting B-cell production of IgE and IgG1. The effect of dupilumab on suppressing IgE synthesis is the presumptive basis for its efficacy in abrogating allergic disease.

Beyond the single-case anecdote described herein, several orthogonal observations suggest a plausible role for the IL-4 signaling axis in the pathogenesis of ITP. First, germline IL-4R polymorphisms have been disproportionately observed in chronic ITP patients and correlate with higher bleeding scores [6]. Second, several IL-4 polymorphisms are associated with greater severity of thrombocytopenia and higher TPO-RA requirements in ITP [6,7]. Third, a recent prospective cohort study in children found that the serum level of IL-4 was significantly higher in those with ITP compared to healthy controls [2,9].

One plausible mechanism of a dupilumab effect on platelet levels might relate to IL-4-mediated effects on Th9 cells [10]. Th9 cells produce significant amounts of IL-9, IL-10, and IL-21 [2,10]. IL-4Rα antagonism by dupilumab could conceivably disrupt Th9 cell activation and reduce IL-21 production, thereby potentially helping to restore Treg/Th17 balance [9,11]. Dupilumab could also hypothetically lower the production of IL-10 by blocking IL-4Rα and reducing the activation of Th9 [4]. Besides Th9 cells involved in the IL-4 pathway, follicular helper T (Tfh) cells secrete IL-4 and IL-21, which is recognized to play a probable role in ITP. This further supports the possible impact of dupilumab on the IL-4 signaling axis that might be contributing to an effect in ITP [3,12,13]. While current data for Th9 and Tfh cells’ involvement in ITP remains limited, and we have no objective metrics of this in our patient, it is an area ripe for deeper immunophenotypic investigation in the future.

Interestingly, in searching the literature for prior associations between and ITP and dupilumab, we found two case reports of patients purported to have *developed* ITP after being treated with the drug for atopic dermatitis [14]. In one case, a patient’s platelets increased to above 500,000 when dupilumab was first initiated but then returned to normal after six months. Roughly one year after the initiation of dupilumab, platelets declined to as low as 5000. The other patient developed Evans Syndrome—with concurrent autoimmune hemolytic anemia (AIHA)and ITP—after starting dupilumab, where platelet count declined to less than 2000 [15]. The first case is particularly curious in that an initial thrombocytosis (e.g., what we observed in our patient) was seen prior to giving way to thrombocytopenia. It is conceivable that manipulation of IL-4 could be one factor contributing to changes in the platelet levels. Moreover, dupilumab has been associated with the emergence of rheumatologic conditions such as inflammatory arthritis and psoriasis, whose possible pathogenesis is also believed to involve the Th17 axis [16,17]. Thus, a deeper molecular understanding of these factors might suggest ways of tipping the response one way or the other, perhaps through supplementation with (or substitution of) other pharmacological upstream or downstream pathway modulators.

In both case studies, ITP developed in the background of underlying atopic dermatitis, an inflammatory immunologic disorder with an established association with ITP [18]. As such, these anecdotes might be an immunologically unique situation. While the patient in this case report has not experienced any side effects of dupilumab as of yet, common side effects reported in clinical trials included injection site reactions, conjunctivitis, upper respiratory tract infections, and arthralgia [19]. The lack of ITP as a reported side effect in the dupilumab registrational Phase 3 trial in patients with various allergic conditions and in post-marketing Phase 4 stage studies suggests it remains quite rare. Furthermore, we were unable to find any reports describing pre-existing ITP being worsened by dupilumab. Both lines of reasoning suggest that prospective studies of dupilumab for treating ITP therefore do not appear to be unduly high risk but would, of course, need to be considered carefully in the context of any interventional study in the future.

Recognizing that association does not equate to causality and that a single patient anecdote is always at risk of being confounded, we spent significant effort looking for obvious alternate explanations besides dupilumab to explicate platelet improvement. We reviewed labs, other medications, clinical notes, and vital signs and interviewed the patient about diet, medication compliance, alcohol and substance use, and other factors and could not find a clear correlation. Nevertheless, in addition to the possibility that we overlooked an objective clue, other factors beyond our ability to practically measure could, of course, be alternate etiologies that must be considered.

ITP is well known to fluctuate, and it is conceivable that the platelet changes seen might have simply been a reflection of this random variation. While the magnitude of the change and the extent of stability before and after the change makes this seem less likely, it is plausible. Similarly, an unrecognized systematic food effect change, potentially an increase in calcium-rich or high-fat foods within 2 h of eltrombopag administration, or interactions with a different level of cations (i.e., calcium, zinc, and magnesium) could alter absorption of eltrombopag, though one would expect this to decrease serum levels to lower its therapeutic effect rather than increase them [19]. A coincidental timing change to medication adherence might also contribute to changes in platelet level. Although we did not see any changes in the patient’s fill history or personal reporting, unless he had been poorly compliant in the past and suddenly improved compliance, this similarly would be expected to move platelets in the opposite direction of that observed. Moreover, since the patient continued a low dose of eltrombopag throughout the above timeline rather than discontinuing it entirely, it is theoretically possible that the apparent improvement following the introduction of dupilumab might be related to some other mechanism of enhancing the response to the existing TPO RA in a certain subset of patients with ITP (e.g., slowing its metabolism) rather than a direct effect on IL-4 signaling.

While this single case report is anecdotal, we view it as sufficiently provocative and hypothesis-generating to alert our colleagues to be on the lookout for similar clinical cases and to call for deeper basic science and translational investigations. The current period of market exclusivity for dupilumab is granted until January 2031 [20]. When this ends, the anticipated improvement in affordability of biosimilars could make dupilumab a more viable alternative, or an option to augment eltrombopag, for patients with ITP if the benefits can be more generally confirmed.

Near-term research directions to further investigate this possibility could include reviewing retrospective analyses of platelet trends in patients with ITP who are taking dupilumab for autoimmune conditions versus a matching ITP-only control group to distinguish spontaneous platelet fluctuation or effects of other therapies. The rarity of this overlap would make this investigation quite challenging in most settings. However, the VA Healthcare System, where this case occurred, serves more than 9.1 million patients annually and would be uniquely well positioned to undertake this natural experiment [21]. A large sample size will help disentangle and characterize other clinical variables or potential biomarkers to predict which specific subsets of patients could most benefit from IL-4Rα antagonists. If ultimately validatable in prospective cohorts, IL-4Rα antagonists or related drugs could prove a useful new therapy class for treatment of ITP.

## Figures and Tables

**Table 1:** Case timeline of relevant events, platelet levels, and eltrombopag dose changes.

Time with Respect to Dupilumab Initiation (T_0_)	Events Description Including Major Clinical Events and Rationale for Eltrombopag Dose Change	Platelet Level (per μL)	Eltrombopag Dose
T − 32 months to T − 12 months	Baseline platelet while on eltrombopag	average 78,000	75 mg daily
T − 10 months	Steroids used for COPD exacerbation and bronchitis	218,000	75 mg daily
T − 9.5 months	2 weeks after steroid use	126,000	75 mg daily
T − 8 months	Benralizumab started		
T − 7.5 months	2 weeks after start of benralizumab	42,000	75 mg daily
T − 6 months	1 month after start of benralizumab	105,000	75 mg daily
T − 2.5 months	5th dose of benralizumab	127,000	75 mg daily
T − 0 months	Dupilumab started		75 mg daily
T + 1 week	1 week after dupilumab started	157,000	75 mg daily
T + 1 month	2 months after dupilumab started	183,000	75 mg daily
T + 3 months	3 months after dupilumab started	181,000	75 mg daily
T + 6 months	6 months after dupilumab started	110,000	75 mg daily
T + 7 months	Eltrombopag dose reduced after several months of sustained platelet levels		50mg daily
T + 9 months	Platelet level remained > 150,000 after 50 mg eltrombopag dose for 2 months, dose reduced to 25 mg daily	158,000	25 mg daily
T + 10 months	Platelet level remained > 150,000 while on 25 mg daily for 1 month, dose was reduced to 12.5 mg	150,000	12.5 mg daily
T + 12 months	One year since starting dupilumab, eltrombopag was reduced to lowest possible dose	71,000	12.5 mg daily
T+ 16 months	Discussed holding eltrombopag, but delayed by pulmonary resection in one month	95,000	12.5 mg daily
T + 17 months	Eltrombopag dose increased for pulmonary resection		25 mg daily
T + 18 months	Eltrombopag dose reduced to 12.5 mg daily after pulmonary resection		12.5 mg daily
T + 18.5 months	2 weeks after eltrombopag back to the lowest dose	95,000	12.5 mg daily
T + 24 months	Taking dupilumab for 2 years	112,000	12.5 mg daily
T + 30 months	Taking dupilumab for 2.5 years	155,000	12.5 mg daily

## Data Availability

The raw data presented in Figure 1 is not included as a supplementary table because of Department of Veterans Affairs privacy policies. Requests to access this data should be directed to the corresponding author.

## Supplementary Materials

The following supporting information can be downloaded at: https://www.mdpi.com/article/10.3390/jcm15197539/s1, Table S1: CARE Checklist of information to include when writing a case report.
