# Supplementary material for: Sustained Platelet Improvement Temporally Associated with Dupilumab in Chronic Immune Thrombocytopenia: A Case Study
Source: J Clin Med. 2026 Sep 28;15(19):7539. doi: 10.3390/jcm15197539 (PMC13665891; doi:10.3390/jcm15197539)
Supplement: Supplementary file 1 [file jcm-15-07539-s001.zip › jcm-4541351-supplementary.pdf]

CARE case report guidelines

CARE Checklist of information to include when writing a case report

(cc) BY-NC-ND

Topic	Item	Checklist item description	Reported on Line
Title	1	The diagnosis or intervention of primary focus followed by the words "case report"	3
Key Words	2	2 to 5 key words that identify diagnoses or interventions in this case report, including "case report"	42
Abstract (no references)	3a	Introduction: What is unique about this case and what does it add to the scientific literature?	23-24
	3b	Main symptoms and/or important clinical findings	28-30
	3c	The main diagnoses, therapeutic interventions, and outcomes	25-28
	3d	Conclusion—What is the main "take-away" lesson(s) from this case?	35-41
Introduction	4	One or two paragraphs summarizing why this case is unique (**may include references**)	69-71
Patient Information	5a	De-identified patient specific information.	73-153
	5b	Primary concerns and symptoms of the patient.	73-76
	5c	Medical, family, and psycho-social history including relevant genetic information	80-82
	5d	Relevant past interventions with outcomes	94-96
Clinical Findings	6	Describe significant physical examination (PE) and important clinical findings.	96-115
Timeline	7	Historical and current information from this episode of care organized as a timeline	table 1, 96-115
Diagnostic Assessment	8a	Diagnostic testing (such as PE, laboratory testing, imaging, surveys).	78-87
	8b	Diagnostic challenges (such as access to testing, financial, or cultural)	87-89
	8c	Diagnosis (including other diagnoses considered)	76-86
	8d	Prognosis (such as staging in oncology) where applicable	N/A
Therapeutic Intervention	9a	Types of therapeutic intervention (such as pharmacologic, surgical, preventive, self-care)	139-145
	9b	Administration of therapeutic intervention (such as dosage, strength, duration)	143-145
	9c	Changes in therapeutic intervention (with rationale)	table 1, 96-115
Follow-up and Outcomes	10a	Clinician and patient-assessed outcomes (if available)	145-146, 214-215
	10b	Important follow-up diagnostic and other test results	130-138
	10c	Intervention adherence and tolerability (How was this assessed?)	126-128, 157-159
	10d	Adverse and unanticipated events	214-217
Discussion	11a	A scientific discussion of the strengths AND limitations associated with this case report	212- 231, 232-248
	11b	Discussion of the relevant medical literature **with references**.	177 - 221
	11c	The scientific rationale for any conclusions (including assessment of possible causes)	177-195
	11d	The primary "take-away" lessons of this case report (without references) in a one paragraph conclusion	256-266
Patient Perspective	12	The patient should share their perspective in one to two paragraphs on the treatment(s) they received	112-115, 145-146, 214
Informed Consent	13	Did the patient give informed consent? Please provide if requested	Yes ☑ No ☐
